# Tree litter functional diversity and nitrogen concentration enhance litter decomposition via changes in earthworm communities

**DOI:** 10.1002/ece3.6474

**Published:** 2020-06-17

**Authors:** Guillaume Patoine, Helge Bruelheide, Josephine Haase, Charles Nock, Niklas Ohlmann, Benjamin Schwarz, Michael Scherer‐Lorenzen, Nico Eisenhauer

**Affiliations:** ^1^ German Centre for Integrative Biodiversity Research (iDiv) Halle‐Jena‐Leipzig Leipzig Germany; ^2^ Institute of Biology Leipzig University Leipzig Germany; ^3^ Institute of Biology/Geobotany and Botanical Garden Martin Luther University Halle‐Wittenberg Halle (Saale) Germany; ^4^ Geobotany Faculty of Biology University of Freiburg Freiburg Germany; ^5^ Department of Renewable Resources Faculty of Agriculture, Life and Environmental Sciences General Services Building University of Alberta Edmonton AB Canada; ^6^ Biometry and Environmental System Analysis Faculty of Environment and Natural Resources University of Freiburg Freiburg Germany

**Keywords:** biodiversity–ecosystem function, BIOTREE, decomposers, litter mass loss, litter traits, macrodetritivores

## Abstract

Biodiversity is a major driver of numerous ecosystem functions. However, consequences of changes in forest biodiversity remain difficult to predict because of limited knowledge about how tree diversity influences ecosystem functions. Litter decomposition is a key process affecting nutrient cycling, productivity, and carbon storage and can be influenced by plant biodiversity. Leaf litter species composition, environmental conditions, and the detritivore community are main components of the decomposition process, but their complex interactions are poorly understood. In this study, we tested the effect of tree functional diversity (FD) on litter decomposition in a field experiment manipulating tree diversity and partitioned the effects of litter physiochemical diversity and the detritivore community. We used litterbags with different mesh sizes to separate the effects of microorganisms and microfauna, mesofauna, and macrofauna and monitored soil fauna using pitfall traps and earthworm extractions. We hypothesized that higher tree litter FD accelerates litter decomposition due to the availability of complementary food components and higher activity of detritivores. Although we did not find direct effects of tree FD on litter decomposition, we identified key litter traits and macrodetritivores that explained part of the process. Litter mass loss was found to decrease with an increase in leaf litter carbon:nitrogen ratio. Moreover, litter mass loss increased with an increasing density of epigeic earthworms, with most pronounced effects in litterbags with a smaller mesh size, indicating indirect effects. Higher litter FD and litter nutrient content were found to increase the density of surface‐dwelling macrofauna and epigeic earthworm biomass. Based on structural equation modeling, we conclude that tree FD has a weak positive effect on soil surface litter decomposition by increasing the density of epigeic earthworms and that litter nitrogen‐related traits play a central role in tree composition effects on soil fauna and decomposition.

## Graphical abstract

We tested the effect of tree functional diversity on litter decomposition in a field experiment manipulating tree diversity and partitioned the effects of litter physiochemical diversity and the detrivore community. We found that litter functional diversity increases soil surface litter decomposition through its effect on epigeic earthworms and that litter nitrogen‐related traits play a central role in tree composition effects on soil fauna and decomposition.

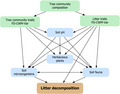

## 1 INTRODUCTION

Biodiversity has been shown to be an important driver of ecosystem functioning (Cardinale, Palmer, & Collins, 2002; Hooper et al., 2005; Loreau & Hector, 2001). As threats to forest species continue to spread and as monospecific tree plantations increase in extent (Paquette & Messier, 2010), it is important to investigate the effects of tree diversity on fauna, herbaceous, and microbial communities that depend on forest habitats, as well as the cumulated impact on crucial ecosystem functions (Cardinale et al., 2012; Hooper et al., 2005). Decomposition is the complementary process to primary production that controls the flow of organic matter and nutrients. As such, it is one of the most critical ecosystem processes that regulates crucial biogeochemical cycles, with consequences for nutrient availability and carbon storage (Krishna & Mohan, 2017; Schlesinger & Bernhardt, 2013). Decomposition results from the activity of microorganisms and detritivorous fauna that break down dead organic matter to gain energy and incorporate matter into their own biomass for growth, maintenance, and reproduction (Bradford, Tordoff, Eggers, Jones, & Newington, 2002; Handa et al., 2014). A diversity of organisms, varying in size and shape, drive decomposition and are structured in complex food webs (Coleman, Crossley, & Hendrix, 2004; Gessner et al., 2010; Hättenschwiler, Tiunov & Scheu, 2005).

Leaf litter is a major resource in forests that feeds into many soil processes and a main carbon and nutrient source for fauna, plants, and microbial communities (Ball, Bradford, Coleman, & Hunter, 2009; Reich et al., 2005). The rate of decomposition of the litter material depends largely on its functional traits, that is, its physical and chemical characteristics that explain variation in decomposition (Nock, Vogt, & Beisner, 2016). For example, high concentrations of elements that are crucial for the performance of detritivores (e.g., N, P, Ca) increase both the palatability of litter and simultaneously its decomposition rate (Cornwell et al., 2008; Freschet, Aerts, & Cornelissen, 2012). In some cases, the ratios of nutrient concentrations can be even more important (e.g., C:N, N:P) than pure concentrations, as they relate better to the stoichiometric needs of the detritivores striving to fulfill their nutritional demands (Hättenschwiler & Gasser, 2005). At the same time, strategies used by trees to protect their leaves from herbivory may lead to traits that inhibit the decomposition of those leaves once abscised. These include physical characteristics associated with the structure of the leaf (e.g., thickness, toughness), as well as chemical characteristics in the form of toxic or repellent secondary compounds (e.g., phenolics, tannins) (Gessner et al., 2010; Ristok, Leppert, Scherer‐Lorenzen, Niklaus, & Bruelheide, 2019; Schindler & Gessner, 2009). Taken together, the palatability of litter material is thus determined by an interplay between different physical and chemical litter traits and their interactions with the decomposer community (Eichenberg, Purschke, Ristok, Wessjohann, & Bruelheide, 2015; Hättenschwiler, Tiunov, & Scheu, 2005).

The impact of the tree community composition on the decomposition process can be twofold. First, the tree community affects litter decomposition by setting the availability and physicochemical properties of the litter material (Handa et al., 2014). In this context, litter species richness was shown to increase decomposition rates in grassland and forest ecosystems in about half of the studied cases, while a third found negative effects (Gartner & Cardon, 2004; Gessner et al., 2010). The main underlying mechanisms that explain these contrasting results include plant species‐specific litter effects on microenvironmental conditions and interactions with the detritivore community (Hättenschwiler et al., 2005). In a litter mixture, the mean tendency of trait values—or community‐weighted mean (CWM; i.e., the mean of trait values for the litter species present in a community)—often is a powerful predictor of decomposition (Bílá et al., 2014). However, it is sometimes also relevant to consider the variance of those trait values, especially in the case of nutrient‐related traits, as it is beneficial for detritivorous organisms to have access to litter materials that differ in nutrient content and other chemical compounds (Barantal, Schimann, Fromin, & Hättenschwiler, 2014; Dudgeon, Ma, & Lam, 1990). In that way, they can fulfill their specific stoichiometric needs by choosing litter species in adequate proportions. When considering multiple traits simultaneously, functional diversity indices (Petchey & Gaston, 2002; FD; Laliberte & Legendre, 2010) provide information on how dissimilar litter species in a mixture are from one another, based on the distribution of trait values, which may have a stronger influence on decomposition processes than species richness (Hättenschwiler & Jørgensen, 2010; Meier & Bowman, 2008). It is therefore expected that higher functional diversity of litter would lead to faster decomposition, as it provides a more diverse resource supply for decomposers, in turn increasing feeding efficiency, both at the individual and community level (Chapman, Newman, Hart, Schweitzer, & Koch, 2013; Finerty et al., 2016; Handa et al., 2014).

**FIGURE 1:**
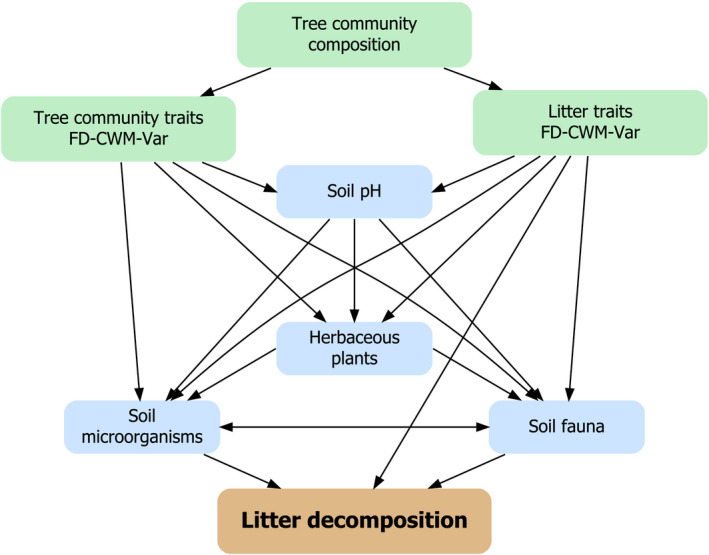
Conceptual model of main components describing the simultaneous pathways in how tree community composition affects litter decomposition. The tree community composition determines the physiochemical traits of the litter and the tree community traits related to life strategies. These trait components—functional diversity (FD), community‐weighted mean (CWM) and variance (Var)—influence soil pH, herbaceous plants, soil fauna, and soil microorganisms, which regulate the environment where decomposition is taking place. Taken together, litter decomposition is the result of the interplay between litter quality, detritivores, and microorganisms

Secondly, tree community composition may influence decomposition by modifying environmental conditions (e.g., soil water retention, light availability, temperature, and soil pH; de Bello et al., 2010; Makkonen, Berg, van Logtestijn, van Hal, & Aerts, 2013) or by affecting the abundance and diversity of other taxa (e.g., invertebrate fauna, microorganisms, herbaceous plants) in the ecosystem that contribute to the decomposition process (Hobbie et al., 2006; Jewell et al., 2017). More diverse tree communities were shown to grow faster (e.g., Huang, 2018) and produce more litter material (Huang et al., 2017; Zhang, Chen, & Reich, 2012), thereby increasing the available resources for detritivores. When considering general traits related to tree life strategies (as opposed to specific litter traits), functionally diverse tree communities create more heterogeneous environments and food sources to support a more complex microbial and faunal community (Cesarz, Fahrenholz, Migge‐Kleian, Platner, & Schaefer, 2007; see Ebeling et al., 2014 for aboveground consumer communities, including soil surface‐dwelling detritivores), and a faster decomposition rate through complementarity effects of a more diverse decomposer community (Gessner et al., 2010; Heemsbergen, 2004; Korboulewsky, Perez, & Chauvat, 2016; de Oliveira, Hättenschwiler, & Handa, 2010). Moreover, tree community composition was also shown to influence herbaceous plants and soil organisms via changes in light availability (Mueller et al., 2016; Williams, Paquette, Cavender‐Bares, Messier, & Reich, 2017). Consequently, there are numerous, nonmutually exclusive pathways by which tree diversity may affect litter decomposition (Figure 1).

In the present study, utilizing a long‐term field experiment manipulating tree functional diversity, we tested the effects of tree litter and community functional diversity on litter decomposition rates, including indirect effects mediated by soil fauna and microorganisms. To do so, we constructed litterbags containing a wide range of leaf litter mixtures that corresponded to the community composition of the experimental plots. We varied the litterbag mesh size to manipulate access to litter by fauna and assess the impact of the macro‐, meso‐, and microfauna and microorganisms separately. We characterized for each experimental plot the soil fauna and microbial communities, herbaceous plant cover, and soil pH as potential explanatory variables of litter mass loss. We expected the exclusion of meso‐ and macrofauna to incrementally reduce litter mass loss in litterbags of smaller mesh sizes. We hypothesized that more functionally diverse litter mixtures would show faster decomposition, with a stronger effect with increasing mesh sizes as macro‐decomposers have been shown to modulate litter diversity effects (Hättenschwiler & Jørgensen, 2010; Patoine et al., 2017) (hypothesis 1). Tree communities with higher functional diversity were expected to provide more diverse food sources and create more complex and heterogeneous environmental conditions that support a more abundant and diverse fauna community (Hooper, 2000), with detritivores in turn contributing to faster litter mass loss (hypothesis 2). In addition to functional diversity effects, we expected the CWM and variance of certain functional traits to affect litter mass loss (Table 1) (hypothesis 3). For instance, as found in a microcosm study using the same litter mixtures (Patoine et al., 2017), more rapid decomposition was expected for mixtures characterized by high nutrient content, low concentration of defensive compounds (tannins and phenolics), and low leaf stability/strength (thickness and toughness). We expected the variance of nutrient‐related traits (leaf N content, leaf C:N, litter C:N) to increase litter mass loss, as diverse mixtures would provide detritivores with complementary food sources to fulfill their nutritional needs (Vos, van Ruijven, Berg, Peeters, & Berendse, 2013). Apart from leaf thickness and toughness, which can also increase litter layer complexity, the same set of traits hypothesized to increase litter palatability was also expected to support a more abundant and diverse soil detritivore community (Hattenschwiler & Gasser, 2005), in turn increasing decomposition rates (Heemsbergen, 2004).

**Table 1:** Results of linear mixed effects models testing the effects of tree and litter FD, the CWM of eight traits, and the variance of three traits on litter mass loss in interaction with litterbag mesh size

	Variable			Mesh size			Interaction			Marg. *R* ^2^
	*χ* ^2^	*df*	*p*‐value	*χ* ^2^	*df*	*p*‐value	*χ* ^2^	*df*	*p*‐value	
Functional dispersion										
Tree community	0.19	1	.663	57.99	2	**<.001**	2.65	2	.266	0.38
Litter	2.38	1	.123	55.25	2	**<.001**	0.36	2	.837	0.40
Trait CWM										
Leaf N	0.95	1	.331	55.56	2	**<.001**	0.99	2	.610	0.38
Leaf C:N	0.79	1	.374	55.55	2	**<.001**	0.93	2	.629	0.37
Litter C:N	6.13	1	**.013**	55.69	2	**<.001**	1.31	2	.519	0.44
SLA	0.97	1	.324	54.62	2	**<.001**	0.04	2	.981	0.36
Leaf phenolics	0.02	1	.891	55.44	2	**<.001**	0.59	2	.746	0.37
Leaf tannins	0.24	1	.626	54.84	2	**<.001**	0.15	2	.928	0.37
Leaf thickness	1.21	1	.272	55.23	2	**<.001**	0.44	2	.804	0.38
Leaf toughness	0.64	1	.424	55.41	2	**<.001**	0.55	2	.759	0.37
Trait variance										
Leaf N	1.10	1	.294	57.34	2	**<.001**	2.43	2	.297	0.39
Leaf C:N	0.01	1	.939	56.74	2	**<.001**	1.57	2	.457	0.37
Litter C:N	2.24	1	.134	55.51	2	**<.001**	0.42	2	.812	0.39

Note

Interaction with mesh size was removed when nonsignificant, but did not affect the *p*‐value of the fixed effect. The values from the full models are presented here. Significance of fixed effects were obtained by Wald chi‐square tests. Significant effects (*p* < .05) are reported in bold.

## 2 MATERIALS AND METHODS

### 2.1 Experimental site

We conducted a litter decomposition field experiment within the tree diversity experiment BIOTREE‐FD (site Bechstedt), described in Scherer‐Lorenzen, Schulze, Don, Schumacher, and Weller (2007). Located in the state of Thuringia, Germany (N 50°54′, E 11°05′, elevation 400–415 m a.s.l), BIOTREE is part of the global tree diversity network TreeDivNet (Paquette et al., 2018; Verheyen et al., 2016). The site BIOTREE‐FD focuses on tree functional diversity effects and consists of 25 hexagonal 43.5 m × 56.0 m plots that are comprised of 44 small circular monospecific subplots. Each subplot was planted in 2003 with 20 trees of one European tree species, and each plot is a mixture of four species. Subplots within plots were included in the design to prevent early competitive exclusion of slow‐growing tree species by fast‐growing ones and are expected to become dominated by one individual canopy over time. Within each monoculture, trees were planted at a spacing of 1 m within rows and 2 m between rows. Tree species for each mixture were chosen from a pool of 16 tree species (Table S1) based on a set of functional traits ranging from leaf chemistry to crown architecture, to create a gradient of functional diversity among the plots. Prior to establishment of the experiment in 2003, the site had been used as pasture and was thoroughly plowed before the experiment was set up. The specific design of BIOTREE‐FD allows us to test the effects of tree functional diversity, while keeping species richness constant. Two of the plots have the same tree species mixture, which is why only one of the two was used in the present study (i.e., 24 plots arranged in four experimental blocks). At the time, this experiment was conducted, the average basal stem diameter per species per plot was (mean ± *SD*) 5.8 ± 2.4 cm, and the crown cover ranged from 60% to 100%.

### 2.2 Litterbags

We collected recently abscised leaves from 13 tree species at the BIOTREE‐FD site in November 2012, and in April 2013 for the remaining three species (*Carpinus betulus*, *Fagus sylvatica*, and *Quercus petraea*), for which abscission is incomplete until spring. The litter material was dried at 60°C for 48 hr to stop the decomposition process. We selected undamaged and noninfected leaves and kept the petioles. To build the litterbags, we used 12 × 12 cm squares of nylon mesh sewn together and filled them using 1 g of leaf material for each of the four tree species in each plot, summing up to a total of 4 g per litterbag. The dried litter was placed in layers following a random order of species. The litter mixture within the bags therefore resembled the naturally occurring litter material in each plot. In order to separate the effects of the macrofauna, mesofauna, and microfauna and microorganisms, we used three different mesh sizes that excluded fauna based on their body size (Coleman, Crossley, & Hendrix, 2004; Decaëns, 2010). First, a 5 mm mesh size allowed the macrofauna, mesofauna, microfauna, and microorganisms to access the tree litter. Second, a 1 mm mesh size only allowed the mesofauna, microfauna, and microorganisms to enter the litterbags. Finally, the smallest mesh size of 0.2 mm constrained the access to only microfauna and microorganisms. Five litterbags per mesh size per plot were used, for a total of 360 litterbags.

The installation of the litterbags at the site took place in June 2013, as soon as possible after all species completed leaf abscission and litterbags were prepared. The litterbags were grouped by three, with one bag of each mesh size, and distributed randomly at the junctions among three within‐plot monospecific subplots to account for heterogeneity within plots. Litterbags in each group were placed in a triangle one meter from each other and held to the ground with metal wires. In April 2015, after an incubation period of 22 months, it was possible to retrieve 167 of the initial 360 litterbags that had been installed. The rest of the litterbags initially installed had either been destroyed or removed from the plots. The high number of disturbed litterbags can likely be attributed to mammal activity (e.g., activity of roe deer). Nevertheless, a sufficient number of litterbags were recovered for each tree functional diversity × mesh size combination, except for one plot and mesh size, where all litterbags were missing. The number of missing litterbags varied among plots and is provided in Table S2.

Once the litterbags were collected, the retrieved litter material was dried (60°C, 48 hr) and stored in a cool (~20°C) and dry room until further processing. We sorted the litter material manually to remove soil aggregates, animal castings, and herbaceous plant material. Due to the advanced state of fragmentation and mixing, we were not able to separate the litter material to species. The dried litter was then cleaned using a fine brush to detach any soil from it. Finally, we sieved the litter with a 2‐mm sieve to separate small litter fragments from sand particles. Long needles passing the sieve were returned to the litter sample, which was then weighed. We assessed litter mass loss (%) as the ratio of the dry weights before and after incubation in the field. As the five bags per mesh size per plot represent pseudoreplicates, we used the average litter mass loss of the litterbags per plot for analysis.

### 2.3 Functional traits

In order to describe the functional characteristics of the tree communities and litter mixtures, we used a total of 14 tree functional traits (Table 1). All continuous trait values, apart from litter C:N, are based on measurements by Hantsch et al. (2014) from fresh leaves sampled on different plots at the BIOTREE‐FD site. The respective trait data are therefore specific to this experimental site. The ordinal traits and litter C:N were used in the design phase of BIOTREE‐FD, before site‐specific trait values could be measured, and are based on literature (Scherer‐Lorenzen, Schulze, et al., 2007). The main trait correlations were observed between leaf phenolics and tannins (Pearson's *r* = 0.94, *p* < .001), leaf N and leaf C:N (*r* = −0.94, *p* < .001), and leaf thickness and toughness (*r* = 0.92, *p* < .001). We calculated the functional dispersion (R package "FD"; Laliberte & Legendre, 2010) of the litter and tree community, respectively. This was done to separately assess the effects of the two groups of traits. Litter functional diversity (litter FD) was calculated from six traits chosen to reflect components that are hypothesized to directly affect the litter decomposition process: leaf type (coniferous or broadleaf), litter C:N ratio, leaf phenolic concentration, leaf tannin concentration, leaf thickness, and leaf toughness. Tree community functional diversity (tree FD) comprises more general traits that describe fundamental aspects of tree life strategies: leaf phenology (evergreen or deciduous), light requirements as adult, mean annual stem growth, crown architecture, root architecture, leaf C:N ratio, and specific leaf area. We considered leaf type and leaf phenology as separate traits due to the presence of the deciduous coniferous tree *Larix decidua* in the species pool. Leaf type governs the leaf physical and chemical aspects, therefore affecting litter decomposition in a more direct way, while leaf phenology is hypothesized to affect decomposition indirectly by defining the rhythm at which litter material enters the system.

Besides the two FD indices, we calculated the community‐weighted mean (Ricotta & Moretti, 2011) and the variance (Laliberte & Legendre, 2010) of specific traits, for which we hypothesized effects on litter mass loss or the soil fauna (see Table S5). Litter FD and trait measures (CWM and variance) related to the litter material considered the relative dominance of species in the litterbags when testing trait effects on the litterbag material. In that case, the weighting had no effect as all litter species were present in equal amounts. However, litter FD and litter trait measures were weighted by average tree species basal area (plot‐specific) when they were related to the litter layer (e.g., effects of litter FD on soil fauna) to represent the different amounts of litter material contributed by trees of different sizes. Tree FD and tree community trait measures were always weighted.

### 2.4 Soil organisms

The surface‐dwelling invertebrate community of each plot was sampled using pitfall traps. The traps were 6 cm in diameter with propylene glycol as conservation agent. In May 2014, we placed one trap in the center of each group of litterbags previously installed (five per plot, 120 in total). A 10 cm plastic roof was installed a few centimeters above each pitfall trap to protect it from rainfall. After 17 days in the field, 114 of the 120 pitfall traps were retrieved. The six missing traps had been disturbed, five of which were from the same plot. The fauna collected was then transferred to 70% ethanol until identification.

We used a stereo microscope and taxonomic keys (Laird‐Hopkins, Bréchet, Trujillo, & Sayer, 2017; Müller, Bährmann, & Köhler, 2015) to measure the 17,033 animals and classify them into 224 morphospecies, based on phylogeny, morphology, and body size. We retrieved all macro‐ and mesofauna specimens (i.e., larger than 0.2 mm in length) from the traps and photographed representative specimens for each morphospecies for later reference. Taxa for which the sampling method was inadequate were excluded from the analysis (e.g., adult Diptera, non‐Formicidae Hymenoptera, some Coleoptera families, Lepidoptera). Soil‐dwelling organisms like earthworms (10 individuals) and diverse larvae were kept in the analysis as they also exhibit epigeic behaviors. We kept 14,925 fauna specimens grouped into 142 morphospecies (Table S3). The data were averaged by plot to avoid pseudoreplication. The biovolume of each morphospecies was calculated using a geometric approximation based on the length, width, and height of the taxon's body, excluding appendages. For each plot, the specimen abundance and morphospecies richness were counted, and biovolume was calculated using a geometric approximation for each morphospecies (Table S4; Farrell, Harpole, Stein, Suding, & Borer, 2015). Biovolumes calculated ranged from 0.006 mm^3^ (oribatid mite) to 2,477 mm^3^ (*Carabus coriaceus*). Morphospecies with fewer than three specimens in total were excluded from the species richness count, in order to reduce the effect of very rare species. Detritivores were identified as taxa that feed at least partially on litter material, following an approach similar to Ebeling, Rzanny, et al. (2018). Scavengers that feed on carrion and exclusive fungal feeders were therefore not included in the detritivore group, as they do not contribute directly to the decomposition of the tree litter material used in this experiment. Abundance, morphospecies richness, and biovolume per plot were also calculated for detritivores, as well as for isopods separately, as they are one of the main litter feeders in temperate forests (Gerlach, Samways, & Pryke, 2013). The average per plot for pitfall fauna abundance was (mean ± *SD*) 654 ± 199, for morphospecies richness 48.6 ± 6.4, and for biovolume 15,663 ± 7,021 cm^3^.

For microbial measurements, three soil cores were collected per plot in November 2012 with a 2 cm diameter corer to a depth of 7 cm. Cores were pooled at the plot level, homogenized, and stored in plastic bags at 4°C until further processing. Basal respiration and microbial biomass carbon were assessed after sieving the samples in the laboratory using a 2 mm mesh. We measured the soil microbial basal respiration and biomass C using an O_2_‐microcompensation apparatus (Scheu, 1992). The basal respiration (µl O_2_ hr^−1^ g^‐1^ soil dry weight) was measured before the addition of substrate as the mean O_2_ consumption rate over 10 hr, once measurements stabilized. The total microbial biomass was measured using substrate‐induced respiration (Anderson & Domsch, 1978), using a glucose solution for forest soil (8 mg/g soil dry weight) added to saturate the soil without creating anaerobic conditions. We used the mean of the lowest three readings within the first 10 hr (but after the initial peak) as the maximum initial respiratory response (MIRR; µl O_2_ hr^−1^ g^−1^ soil dry weight). Microbial biomass (µg C g^−1^ soil dry weight) was calculated as 38 × MIRR (Beck et al., 1997).

As earthworms are a crucial detritivore group that was poorly sampled by pitfall traps, we supplemented our study with earthworm abundance and biomass data collected using the mustard method from a related study conducted in spring 2013 by Schwarz et al. (2015). Specifically, three spatial replicates of 0.25 m^2^ each were taken per plot using hand sorting and subsequent mustard solution extraction in the dug holes (10 L per replicate, 10 g of mustard powder per L). Earthworms were identified to species and grouped to ecological groups (anecic, epigeic, and endogeic). We used the abundance and biomass of total earthworms, as well as for ecological groups separately, as additional soil fauna variables. The list of earthworm species present at the site, as well as sampling methodologies, are described in Schwarz et al. (2015). Because of the difference in sampling methods and measures (i.e., biomass versus biovolume), earthworm data were analyzed separately and was not grouped with the pitfall fauna.

### 2.5 Other explanatory variables

Soil pH was measured from the retrieved soil cores using 10 g of air‐dried soil and 25 ml 1 M CaCl_2_ solution. We used plant cover data taken from a herbaceous vegetation survey conducted in summer 2015 (Ohlmann, 2016). Each plot was surveyed at 16 locations, where understory plant species were identified, and total herbaceous cover was estimated. We used the mean value of the herbaceous cover for each plot. Canopy leaf area index was also measured for each plot at 80 cm of height using a LICOR LAI Plant Canopy Analyzer. As herbaceous cover highly correlated to leaf area index (*R* ^2^ = 0.76, *p* < .001), we only used herbaceous cover in our analyses.

### 2.6 Statistical analyses

We first tested the effect of mesh size on litter mass loss, using a linear mixed effect model with plot as random term (LMMs; R package “lme4”) and a post hoc Tukey's HSD test. We then separately tested the effects of litter FD, tree FD, 11 tree trait metrics (CWM or variance, see Table 1 and Table S5), 17 soil fauna variables, soil basal respiration and microbial biomass, herbaceous cover, and soil pH on litter mass loss (Table 2). We analyzed the effects of these explanatory variables on litter mass loss always in interaction with litterbag mesh size by fitting LMMs with plot nested in block as random factor. Block was removed from the model, as it did not explain any variance, and its removal did not change the models significantly (ANOVA, all *p* > .95). Only plot was kept as random term. We also tested the effects of the pitfall fauna by restricting the considered species to those able to enter the litterbags (body width < mesh size) for the 5 mm and 1 mm mesh sizes, to test for litter mass loss in specific mesh size bags. To assess how the soil fauna was affected, we analyzed the effects of litter FD, tree FD, 9 tree trait metrics, soil basal respiration and microbial biomass, herbaceous cover and soil pH on the 17 soil fauna variables (Table S6) using LMMs with block as random factor, as we only had one sample per plot. Finally, we tested the effects of litter FD and tree FD on soil basal respiration and microbial biomass, herbaceous cover, and soil pH using LMMs with block as random factor. We did not correct for multiple statistical tests considering the mathematical and logical reasoning by Moran (2003), which presents arguments against the sequential Bonferroni adjustment, but caution the reader that a high number of models were tested. We used the R package “car” to retrieve chi‐square values and *p*‐values for LMMs (Fox & Weisberg, 2011). Marginal *R* ^2^ values, that is, the fraction of the variances explained by fixed factors, were calculated from the package “MuMIn” (Nakagawa & Schielzeth, 2013).

**Table 2:** Results of linear mixed effects models testing the effects of the abundance, morphospecies richness, and biovolume of total fauna, detritivores and isopods from pitfall traps, and the abundance and biomass of all earthworms (total) and anecic, epigeic, and endogeic earthworms separately, soil basal respiration and microbial biomass, herbaceous cover, and soil pH on litter mass loss in interaction with litterbag mesh size

	Variable			Mesh size			Interaction			Marg. *R* ^2^
	*χ* ^2^	*df*	*p*‐value	*χ* ^2^	*df*	*p*‐value	*χ* ^2^	*df*	*p*‐value	
Total fauna										
Abundance	0.15	1	.700	53.34	2	**<.001**	1.09	2	.580	0.37
Richness	0.61	1	.436	53.92	2	**<.001**	1.53	2	.467	0.38
Biovolume	0.43	1	.510	54.76	2	**<.001**	2.38	2	.305	0.38
Detritivores										
Abundance	0.01	1	.927	54.13	2	**<.001**	1.60	2	.449	0.37
Richness	0.02	1	.881	52.17	2	**<.001**	0.12	2	.943	0.36
Biovolume	0.69	1	.405	54.40	2	**<.001**	1.71	2	.425	0.38
Isopods										
Abundance	0.13	1	.716	57.48	2	**<.001**	4.17	2	.124	0.39
Richness	0.14	1	.712	57.39	2	**<.001**	4.39	2	.111	0.39
Biovolume	0.05	1	.819	56.92	2	**<.001**	3.76	2	.152	0.39
Total earthworms										
Abundance	0.08	1	.778	56.57	2	**<.001**	1.56	2	.459	0.37
Biomass	0.23	1	.631	57.84	2	**<.001**	2.29	2	.318	0.38
Anecic earthworms										
Abundance	0.66	1	.418	61.65	2	**<.001**	5.14	2	.077	0.40
Biomass	0.50	1	.480	63.53	2	**<.001**	6.72	2	**.035**	0.41
Epigeic earthworms										
Abundance	4.90	1	**.027**	60.92	2	**<.001**	4.97	2	.084	0.45
Biomass	5.67	1	**.017**	62.89	2	**<.001**	6.43	2	**.040**	0.47
Endogeic earthworms										
Abundance	0.07	1	.795	58.50	2	**<.001**	3.05	2	.218	0.38
Biomass	0.62	1	.431	59.87	2	**<.001**	4.09	2	.130	0.40
Soil micro organisms										
Basal respiration	0.02	1	.891	48.98	2	**<.001**	0.06	2	.971	0.36
Biomass C	0.04	1	.852	51.26	2	**<.001**	1.90	2	.386	0.37
Herb layer										
Herbaceous cover (%)	1.77	1	.183	55.19	2	**<.001**	0.57	2	.751	0.39
Soil										
pH	0.21	1	.650	61.12	2	**<.001**	1.76	2	.414	0.41

Note

Interaction with mesh size was removed when nonsignificant, but did not affect the *p*‐value of the fixed effect. The values from the full model are presented here. Significance of fixed effects were obtained by Wald chi‐square tests. Significant effects (*p* < .05) are reported in bold.

We used the main significant relationships found in LMMs to build a structural equation model (*SEM*) describing direct and indirect effects on litter mass loss, by separating the different mesh sizes into separate variables. The structure of this *SEM* was based on our conceptual model (Figure 1). We used the R package “lavaan” (Rosseel, 2012) to extract path coefficients. All statistical analyses were performed using the statistical software R ver. 3.6 (R Core Team, 2019).

## 3 RESULTS

### 3.1 Litter mass loss

**FIGURE 2:**
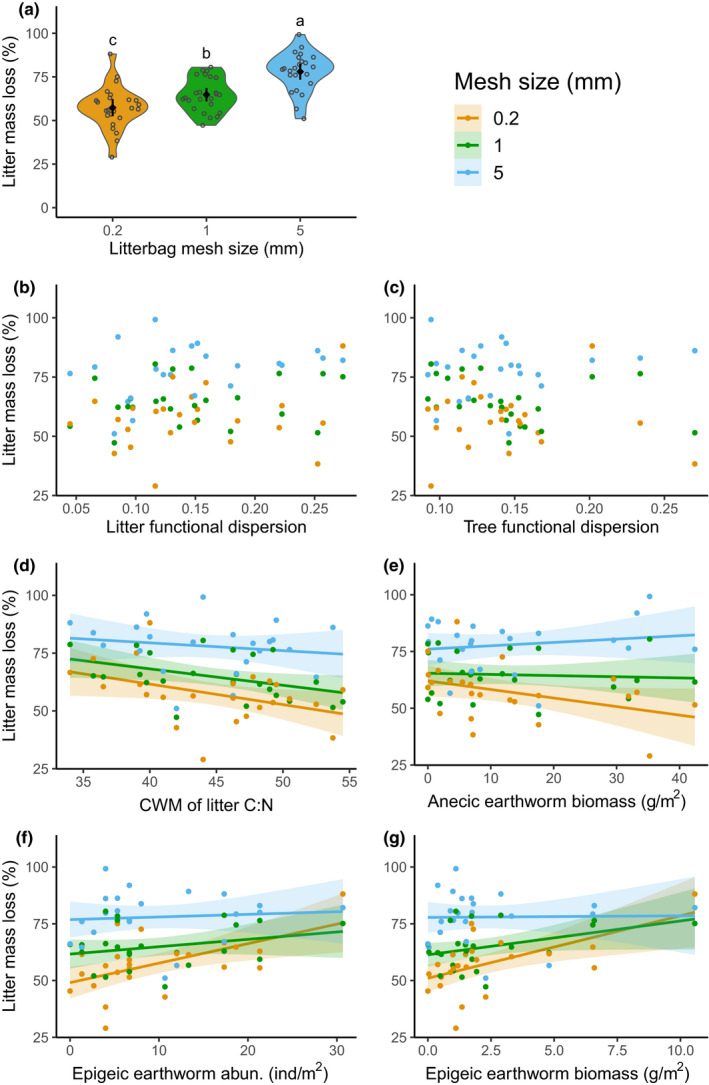
(a) Effect of litterbag mesh size (in mm) on litter mass loss (black symbols are means ± 95% confidence intervals, *χ* ^2^ (2) = 53.0, *p* < .001). Gray circles show jittered data points. Different letters indicate significant differences among litterbag mesh sizes (Tukey's HSD test). Relationships between litter mass loss and (b) litter functional dispersion, (c) tree functional dispersion, (d) CWM of litter C:N, (e) anecic earthworm biomass (f) epigeic earthworm abundance (abun.), and (g) epigeic earthworm biomass (Table 2). Lines are based on linear regressions with 95% confidence. No regression line is shown when no significant effect was found

Litter mass loss was incrementally higher in litterbags with larger mesh sizes (*χ* ^2^ (2) = 53.0, *p* < .001, Figure 2a), with a loss in the 0.2 mm, 1 mm, and 5 mm mesh bags of (mean ± *SE*) 57.3 ± 2.5%, 64.8 ± 2.0%, and 78.0 ± 2.3%, respectively. We did not find any significant effect of litter FD and tree FD on litter mass loss (Figure 2b,c, Table 1). From the nine trait CWMs tested, only litter C:N ratio significantly affected litter mass loss, with a decrease of litter mass loss for higher C:N values; this relationship was consistent across mesh sizes (Figure 2d, no significant interaction effect in Table 1). The variance of nutrient content was not found to significantly influence litter mass loss for any of the three N‐related measures (Table 1).

Litter mass loss was also not significantly influenced by the abundance, morphospecies richness, nor biovolume of any of the pitfall groups tested (Table 2), even after controlling for invertebrate size to only consider taxa that could fit into the litterbags for the 5 mm and 1 mm mesh sizes (all *p* > .05, not presented). Anecic earthworm abundance was not significantly correlated to litter mass loss (Table 2), but anecic earthworm biomass reduced litter mass loss in the smallest mesh size, had a weaker effect in the 1 mm mesh size, and a weak positive effect on litter mass loss in the 5 mm mesh size (Figure 2e, Table 2). Epigeic earthworm abundance was significantly positively correlated to litter mass loss irrespective of mesh size (Figure 2f, Table 2), while the biomass of epigeic earthworms interacted with mesh size in a way that the positive earthworm effect on litter mass loss was stronger for smaller mesh sizes and almost inexistent for the 5 mm mesh size (Figure 2g, Table 2). Endogeic earthworm abundance and biomass were not found to affect litter mass loss. Soil basal respiration, microbial biomass, herbaceous cover, and soil pH were not significantly correlated with litter mass loss (all *p* > .05, Table 2).

### 3.2 Soil fauna

Of the 255 models tested on soil fauna variables, 19 had significant results (Table S6). The statistical results of significant models (*p* < .05) are presented in more detail below and in Table 3. When both models on abundance and biovolume (or biomass) of a group were significant and the variables were strongly correlated, only abundance is shown (Appendix 1: Figures [Link], [Link], [Link]). Tree community FD had no significant effect on any of the soil fauna variables, but litter FD showed positive effects on epigeic earthworm abundance and biomass, as described below. The effects of tree traits and environmental variables on soil fauna are described for each group separately. The pitfall fauna abundance was significantly positively correlated to soil pH (Figure S3c) and was negatively correlated to basal respiration (Figure S3a). Pitfall fauna biovolume was positively correlated with the CWM of leaf N (Figure S1a) and negatively with the CWM of leaf C:N (not shown as leaf N and leaf C:N are highly negatively correlated), and herbaceous cover (Figure S2a). Further analysis showed that average pitfall fauna biovolume decreased with herbaceous cover (*χ* ^2^ (1) = 7.25, *p* = .007, Figure S2b). Pitfall fauna morphospecies richness also decreased with the CWM of leaf C:N (Figure S1b).

**Table 3:** Results of linear mixed effects models testing the effects of litter and tree functional dispersion (FD), the CWM of six traits, the variance (Var) of three traits, soil basal respiration and microbial biomass, herbaceous cover and soil pH on the abundance, morphospecies richness, and biovolume of total fauna, detritivores and isopods from pitfall traps, the abundance and biomass of all earthworms (total), and epigeic, anecic, and endogeic earthworms separately

Response variable	Explanatory variable	*df*	*χ* ^2^	*p*‐value	Marginal *R* ^2^	Effect
Total fauna						
Abundance	Soil basal respiration	1	5.76	.016	0.224	↘
	Soil pH	1	11.63	.001	0.356	↗
Biovolume	CWM of leaf N	1	7.23	.007	0.159	↗
	CWM of leaf C:N	1	6.10	.014	0.143	↘
	Herbaceous cover	1	5.18	.023	0.110	↘
Richness	CWM of leaf C:N	1	3.89	.049	0.150	↘
Detritivores						
Abundance	Soil pH	1	6.85	.009	0.202	↗
Biovolume	Soil pH	1	4.09	.043	0.125	↗
Richness	CWM of litter C:N	1	10.15	.001	0.223	↘
Total earthworms						
Abundance	Herbaceous cover	1	3.90	.048	0.138	↗
Biomass	Var of litter C:N	1	4.39	.036	0.160	↘
Anecic earthworms						
Abundance	Var of litter C:N	1	6.60	.010	0.223	↘
Biomass	Var of litter C:N	1	6.27	.012	0.214	↘
Epigeic earthworms						
Abundance	Litter FD	1	4.01	.045	0.148	↗
	CWM of SLA	1	4.78	.029	0.172	↘
Biomass	Litter FD	1	5.40	.020	0.190	↗
	CWM of SLA	1	7.95	.005	0.257	↘
	CWM of phenolics	1	4.42	.036	0.161	↘
Endogeic earthworms						
Biomass	Soil microbial biomass	1	4.87	.027	0.188	↗

Note

We used experimental block as random factor. Only significant effects (*p* < .05) are reported in the table. Significance of fixed effects were obtained by Wald chi‐square tests. The degrees of freedom (*df*), chi‐square, and goodness of fit (marginal *R* ^2^) are also provided. Effect is shown for positive (↗) and negative (↘) relationships.

**FIGURE 3:**
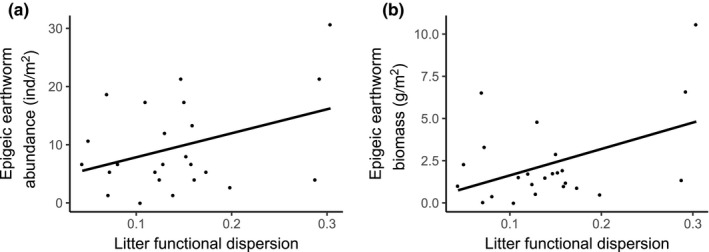
Influence of litter functional dispersion on epigeic earthworm (a) abundance and (b) biomass. Lines are based on linear regressions

Pitfall detritivore abundance and biovolume increased with increasing soil pH (Figure S3d, detritivore biovolume not shown). Detritivore richness was negatively correlated with the variance of litter C:N (Figure S1c). The total earthworm abundance increased with herbaceous cover (Figure S2c) and decreased with the variance of litter C:N (Figure S1d). Anecic earthworms, which accounted for 45% of the total earthworm biomass on average per plot, also had their abundance and biomass negatively correlated to the variance of litter C:N (Figure S1e, biomass not shown). Epigeic earthworm accounted for 13% of the total earthworm biomass, and their abundance and biomass responded positively to increasing litter FD (Figure 3) and negatively to CWM of leaf SLA (Figure S1f, biomass not shown). The epigeic earthworm biomass was also negatively correlated to the CWM of leaf phenolics (Figure S1g). Finally, endogeic earthworm biomass was positively correlated to soil microbial biomass (Figure S3b). When considering other pathways, litter and tree community FD had no significant effects on soil basal respiration, microbial biomass, herbaceous cover, and soil pH (all *p* > .05).

### 3.3 Structural equation modeling

**FIGURE 4:**
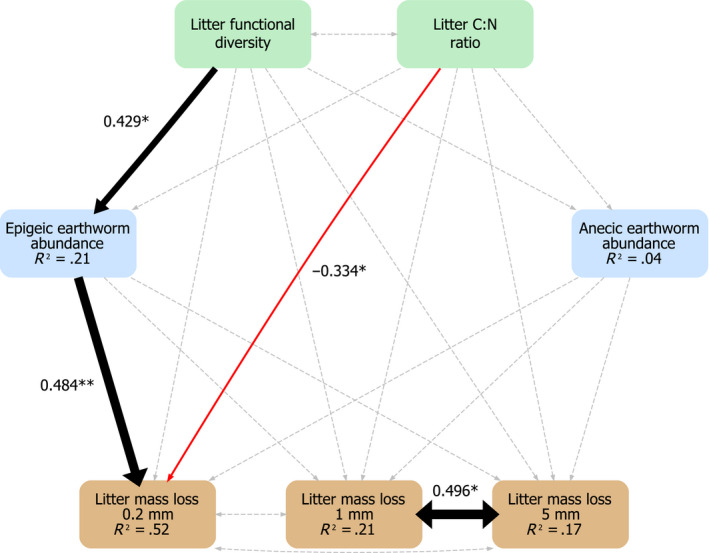
Structural equation model including all direct and indirect pathways from litter functional diversity (FD) and litter C:N ratio to litter mass loss (LML) in litterbags of three different mesh sizes via the epigeic and anecic earthworm (EW) abundances, as the main tree community aspects and fauna groups influencing LML. Black and red lines represent significant (*p* < .05) positive and negative relationships, respectively, with the strength shown by arrow width. Gray dashed line are nonsignificant relationships. Double‐headed arrows are for correlations. Numbers on arrows are standardized path coefficients (see Table S7)

Based on our conceptual model (Figure 1) and the main significant relationships found with LMMs, we constructed an *SEM* to compare direct and indirect effects of functional diversity and tree traits on litter mass loss through the soil fauna community. Our model used litter FD and litter C:N with effects on epigeic and anecic earthworm abundance and all direct and indirect paths to affect litter mass loss in the three mesh sizes (Figure 4; Table S7). We assumed litter FD and litter C:N as well as litter mass loss in the different mesh sizes to be correlated. Our hypothesized *SEM* could not be rejected as a potential explanation of the observed covariance matrix (Test statistic = 0.001, *p*‐value = 0.972, *df* = 1, Figure 4). The *SEM* shows that litter mass loss in the litterbags with the smallest mesh size increased with increasing litter FD via a significant increase in epigeic earthworm abundance and was negatively related to the CWM of litter C:N.

## 4 DISCUSSION

Using a decade‐old tree diversity experiment and comprehensive data on the soil fauna and microbial community, soil pH, and the understory plant community, we show in this study that litter mass loss increased with increasing mesh size of the litter bags, confirming that meso‐ and macrodetritivores play a central role in this process (Hattenschwiler & Gasser, 2005). While weak litter functional diversity effects on surface litter mass loss provided little support for our hypothesis (a), we observed that these were indeed mediated by epigeic earthworms, confirming our hypothesis (b), that detritivores contribute to litter diversity effects. Further analyses suggest that litter traits related to nutrient concentrations are of particular relevance for these observed relationships and mass loss in general, supporting our hypothesis (c).

We attributed the increase in litter mass loss in relation to increasing mesh size to the respective fauna having access to the litter material. While the size of the mesh size may create differential microclimatic effects (Bradford et al., 2002), these effects are usually small compared to that of fauna access (Bokhorst & Wardle, 2013). The size of litter fragments that could fall or be extracted from the bag was also defined by the mesh size, and some of the results observed might be partly attributed to that. Nevertheless, the reader should note that such potential artifacts of the experimental approach cannot be fully excluded. In addition, we took all possible measures to minimize this effect by handling the litterbags carefully at every step of the study. In addition, by sieving the remaining litter with a 2 mm mesh size, we partially accounted for this effect between litterbags with 1 mm and 0.2 mm mesh sizes, and still showed an important litter mass loss difference (Figure 2a).

While there is evidence for positive effects of plant functional diversity on litter decomposition rates in grassland biodiversity experiments (e.g., Scherer‐Lorenzen, 2008), only a limited number of studies has tested the effect of tree FD on litter decomposition thus far (Hättenschwiler et al., 2005), with mostly nonsignificant results. Using the original FD (FD_orig_) index calculated during the setup of the BIOTREE‐FD experiment (Scherer‐Lorenzen, Schulze, et al., 2007) Schwarz et al. (2015) did not find any significant effect of FD_orig_ on earthworm abundance and biomass. FD_orig_ has a lot of trait overlap with our tree community FD (leaf phenology, light requirements as adults, mean annual stem growth, crown architecture, root architecture, and specific leaf area), which was also not found to affect litter mass loss, soil fauna, and microorganisms in the present study. It is very likely that tree FD influences other ecosystem functions and taxa (e.g., fungi, aboveground invertebrate herbivores, vertebrates), but its effect on the decomposition process may be minor. In one study in a natural forest, decomposition rate was not affected by tree FD, nor by species richness of the plot tree community (Fujii et al., 2017). In another tree diversity experiment using gradients of species richness and functional diversity on experimental plots, five FD indices were calculated using litter chemical traits, leaf physical traits, all litter and leaf traits, growth and structural traits, and root traits, respectively (Jewell et al., 2017). Despite using indices based on diverse sets of potentially relevant traits, no significant litter FD or tree FD effects were found on litter decomposition (Jewell et al., 2017). Similarly, a few other studies also found that FD effects on litter decomposition in mixtures were not significant (Frainer, Moretti, Xu, & Gessner, 2015; Tardif & Shipley, 2015). Partly, inconsistent findings may be due to different litter traits being considered in previous studies as well as temporal dynamics in the decomposition process, and the varying role of litter traits during different stages of litter decomposition (Patoine et al., 2017; Ristok et al., 2017). To exemplify these interactions, a laboratory study that used the same litter mixtures as presented here (i.e., based on the BIOTREE‐FD site design) found a positive effect of litter FD on decomposition, but only for a given time period of the experiment, with the effect disappearing at later stages of the experiment (Patoine et al., 2017). It is therefore possible that earthworm abundance also affected litter mass loss in the 1 mm and 5 mm litterbags, but that this effect was not visible anymore due to a too advanced stage of decomposition. The more advanced decomposition phase in litterbags with larger mesh sizes may also be a reason why we were able to explain much more variance of litter mass loss in the smallest mesh size litter bags (>50%) in comparison with the larger mesh sizes (~20%; Figure 4).

Our present results confirm that litter quality is a significant driver of litter decomposition (Hättenschwiler et al., 2005). More specifically, we found that the CWM of litter C:N was a significant trait predictor of litter mass loss, similar to results from other studies (e.g., Scherer‐Lorenzen, Bonilla, et al., 2007). A high C:N ratio represents a low nutrient density, and thus, poor food quality for detritivores, and is therefore correlated with a low litter mass loss in the litterbags, as we hypothesized. N‐related traits are often strong indicators of litter mass loss as nitrogen plays a key role in decomposition processes, and N‐rich food sources are crucial nutritional inputs for detritivores (e.g., Martinson et al., 2008; Milcu, Partsch, Scherber, Weisser, & Scheu, 2008). The finding that the effect of C:N was similar for litterbags of all mesh sizes may indicate the importance of N for decomposer and detritivore taxa of all sizes, from microorganisms to macrofauna. The experimental design of BIOTREE‐FD with a low number of plots (24 plots) may limit our power to find the expected effects from other litter traits on litter mass loss though. In a related laboratory experiment (Patoine et al., 2017), leaf N, but also leaf phenolics, tannins, thickness, and toughness were found to correlate with litter mass loss, but only when tree species‐specific litter mass loss was considered, which was not possible to assess in the present experiment, as the remaining litter material could not be sorted to species, due to the advanced fragmentation. While it is technically challenging (or even impossible in long‐term experiments) to identify decomposed litter material to tree species, species‐specific litter decomposition measures in mixtures are highly valuable to provide mechanistic information of detritivore food choices.

Considering indirect pathways from tree FD to litter mass loss via the soil fauna community, microorganisms, soil pH, and the understory vegetation provided additional insights into the drivers of decomposition rates. Such an approach was also recently used to show indirect effects of plant functional diversity on belowground processes in a forest ecosystem, while direct effects were not observed (Fujii et al., 2017). We expected the soil fauna, especially detritivores, to explain FD effects on litter mass loss. Partly confirming this hypothesis, densities of epigeic earthworms significantly increased litter mass loss. However, in contrast to our expectation, epigeic earthworm density had a stronger positive effect with litterbags of smaller mesh sizes, where the fauna had no direct access to the litter material. Anecic earthworm biomass only had a weak positive effect on litter mass loss in 5 mm mesh bags, and a stronger negative effect in 0.2 mm mesh bags. Earthworms represent some of the key detritivore taxa in many ecosystems (Edwards, 2004), and anecic earthworms especially have a strong effect on soil physical properties and microbial communities (Brown, 1995; Eisenhauer, 2010). The mismatch between mesh size and the earthworm effect that we observed on litter mass loss might suggest that the effect seen in the litterbags was not due to direct consumption, fragmentation, and/or burial by earthworms, but rather to a change of the litter microbial community by inoculation of the litter material with earthworm castings and mucus (Holdsworth, Frelich, & Reich, 2008; Medina‐Sauza et al., 2019; Nechitaylo et al., 2010). This speculation is supported by the observation that many earthworm castings were found in and around litter bags. Accordingly, a previous study found that soil fauna can affect the decomposition rate in litterbags where they have no access by changing moisture patterns and influencing the bacterial and fungal community (Bradford et al., 2002). It is therefore important to note that the exclusion of fauna using a fine mesh prevents consumption, but does not remove other indirect effects. In this experiment, the indirect effect of earthworms might have been countered in larger litterbag mesh sizes by the presence of other fauna that disturbed microorganisms in the litterbags; but this speculation needs to be addressed in future studies. Thus, some macrofauna groups may also be important drivers of decomposition, even where direct access to the litter is constrained (Holdsworth et al., 2008), like in decomposition experiments using teabags (Djukic et al., 2018; Keuskamp, Dingemans, Lehtinen, Sarneel, & Hefting, 2013).

Plots with a higher litter FD were found to have a significantly higher epigeic earthworm abundance and biomass, in accordance to a previous study (De Wandeler et al., 2018). By creating a more complex litter layer, litter FD increases habitat heterogeneity and food sources for the fauna that live in it (Hooper, 2000). Epigeic earthworms especially only dwell on the soil surface (Brown, 1995) and may thus benefit from a thicker and more complex litter layer. Earthworm communities have been shown to profit from a more rich and diverse litter material, which can increase their population density (Cesarz et al., 2007). Litter FD, however, did not influence any of the fauna groups in the pitfall samples, which we expected to respond strongly to changes in litter quality (like detritivores). Similar results were recently found in a grassland experiment, where ground‐dwelling invertebrates, and especially detritivores, reacted less strongly to changes in plant species richness than vegetation‐associated invertebrates, such as herbivores and predators (Ebeling, Hines, et al., 2018; Ebeling, Rzanny, et al., 2018), although earthworms were not included in that analysis. Ebeling, Rzanny, et al. (2018) discussed that while detritivores have preferences in terms of litter material, many of them can feed on a diversity of substrates, which may partially explain that there was no effect of plant species richness on detritivore species richness and that detritivores were instead mostly affected by plant functional groups like the presence of N‐fixing legumes (Eisenhauer et al., 2009; Milcu et al., 2008). However, still in the same experiment (Ebeling, Hines, et al., 2018), isopod abundance was shown to increase with plant species richness, possibly caused by higher plant biomass and more diverse food sources available (Ebeling et al., 2014). In our case, isopods did not respond significantly to any of the explanatory variables we tested.

Nutrient availability from the litter content is an important predictor of the fauna community. In our study, a number of pitfall fauna groups were affected by the CWM of N‐related traits: a high N content (or low C:N) had a positive effect on fauna biovolume and richness, as well as on detritivore richness. Nutrient content is a crucial trait for plant species that influence the palatability of the litter material for detritivores (Aber, Melillo, & McClaugherty, 1990; Cadish & Giller, 1997; Coûteaux, Bottner, & Berg, 1995). Some taxa may benefit more from N‐rich litter materials, while others have more complex nutrient requirements, in which case a more diverse diet may be necessary (Hattenschwiler & Gasser, 2005). In contrast to our expectations, anecic earthworm abundance and biomass decreased with a higher litter C:N variance, which we are not able to explain. We can only speculate that other traits may have covaried, such as chemical compounds that decelerate decomposition and decreased the performance of anecic earthworms. The consideration of additional leaf and litter nutrients (e.g., Ca, Mg, K) may have provided higher explanatory power is this study (Desie et al., 2020; Reich et al., 2005) and could be included in future studies. While phenolic concentration had a negative effect on soil fauna as we expected, as observed for epigeic earthworm biomass, the negative effect of SLA on epigeic earthworm abundance and biomass is contrary to our hypothesis. While a higher SLA represents a thinner or less dense leaf material, it may not necessarily translate into more palatable leaf material. Other leaf structural compounds (e.g., lignin, hemicellulose) might have been better suited to represent decomposition‐relevant traits.

Besides litter composition, soil pH, the microbial community, and herbaceous cover were also found to affect the soil fauna community (De Wandeler et al., 2016), sometimes with a stronger effect than that of other variables. Soil pH at the site varied substantially between 5.3 and 7.1, and was positively correlated to fauna variables in three cases (ground‐dwelling fauna abundance, and detritivore abundance and biovolume). Low pH values represent acidic soil conditions that are generally less favorable for soil fauna (Lavelle, Chauvel, & Fragoso, 1995; Staaf, 1987). Soil pH was not affected significantly by FD or CWM of any trait (all *p* > .05) and had no significant effect on earthworms at the study site (Schwarz et al., 2015). Thus, pH effects may have been mainly due to general pH differences among plots that are independent of the tree communities.

Ground‐dwelling fauna biovolume was found to decrease with herbaceous cover, and we showed that this is related to a decrease of the average individual size (Figure S2). While light availability and herbaceous cover were previously found to have positive effects on fauna diversity (Mueller et al., 2016), we observed that the presence of a dense herbaceous layer hinders, if not the presence, at least the activity of larger invertebrate taxa (Eisenhauer, Milcu, Sabais, & Scheu, 2008), which may lead to a shift toward smaller taxa in the traps. Thus, future studies should investigate such potential “barrier effects” of a dense vegetation and how important they are in comparison with other indirect light effects, such as through soil temperature (Mueller et al., 2016).

Interestingly, we found a significant correlation of soil microbial biomass and endogeic earthworm biomass. Earthworms are known to change the soil structure and microbial community composition, for example, by transforming and incorporating organic matter in the soil and altering soil aeration and water content (Edwards, 2004; Eisenhauer, 2010). In turn, some soil microorganisms and/or their exoenzymes may play an important role for earthworm nutrition (e.g., Bonkowski, Griffiths, & Ritz, 2000). Thus, the observed positive relationship between soil microbial biomass and endogeic earthworms may be due to these potential reciprocal effects, as endogeic earthworms are mostly active in the top 30 cm of the soil in the study region. Future studies should explore how belowground decomposition processes are influenced by abiotic and biotic drivers along functional tree diversity gradients, and if interactions between endogeic earthworms and soil microorganisms play a significant role.

Considering that the trees from BIOTREE‐FD were only planted for about 10 years on a former agricultural site at the moment of the study, it is possible that tree functional diversity effects would only become visible after a longer period of time, as complementarity effects are stronger in older multi‐aged forests (Leuschner, Jungkunst, & Fleck, 2009) and may increase over time (Guerrero‐Ramírez et al., 2017). Accordingly, multiple grassland experiments have shown that plant diversity effects on soil organisms increase over time (Thakur et al., 2015; e.g., Strecker, Macé, Scheu, & Eisenhauer, 2016). In addition, this study used site‐specific plant trait measurements to account for the site's geoclimatic conditions, but not for plot variability and thus for intraspecific variability and trait plasticity. In future studies, plot‐specific trait measurements would allow to assess the resulting effects of community composition on ecosystem functions through trait plasticity (Freschet, Bellingham, Lyver, Bonner, & Wardle, 2013; Sack, Melcher, Liu, Middleton, & Pardee, 2006).

In conclusion, we here provide a mechanistic link between litter traits and decomposition by showing that litter FD has an influence on litter decomposition by increasing epigeic earthworm populations. This effect is however not sufficiently strong to observe a direct effect of litter FD on decomposition. In addition, our findings suggest that certain soil fauna groups can be promoted by management decisions that increase the functional diversity of tree mixtures, arguing for an increase of tree diversity in managed forests, which was also shown to support a broader set of ecosystem functions (van der Plas et al., 2016). Especially, litter traits related to high nutritional value, such as N concentration and its variability, were found to play an important role. These findings suggest that to understand decomposition across environmental contexts, it is important to assess the main biotic and abiotic drivers of this process as well as the nutritional limitations of the decomposers.

## CONFLICT OF INTEREST

No conflict of interest to declare.

## AUTHOR CONTRIBUTIONS

**Guillaume Patoine:** Conceptualization (equal); data curation (equal); formal analysis (lead); investigation (lead); methodology (equal); software (lead); validation (equal); visualization (lead); writing–original draft (lead); writing–review and editing (lead). **Helge Bruelheide:** Data curation (equal); methodology (supporting); project administration (equal); supervision (supporting); writing–original draft (supporting); writing–review and editing (supporting). **Josephine Haase:** Data curation (supporting); investigation (supporting); writing–original draft (supporting); writing–review and editing (supporting). **Charles Nock:** Conceptualization (supporting); data curation (equal); project administration (supporting); resources (equal); supervision (supporting); validation (supporting); writing–original draft (equal); writing–review and editing (equal). **Niklas Ohlmann:** Investigation (supporting); writing–original draft (supporting); writing–review and editing (supporting). **Benjamin Schwarz:** Conceptualization (supporting); data curation (equal); formal analysis (supporting); investigation (supporting); validation (supporting); writing–original draft (supporting); writing–review and editing (supporting). **Michael Scherer‐Lorenzen:** Conceptualization (supporting); data curation (supporting); funding acquisition (supporting); project administration (equal); resources (equal); supervision (supporting); validation (supporting); writing–original draft (supporting); writing–review and editing (supporting). **Nico Eisenhauer:** Conceptualization (equal); data curation (equal); formal analysis (equal); funding acquisition (equal); investigation (equal); methodology (equal); project administration (equal); resources (equal); supervision (lead); validation (equal); writing–original draft (equal); writing–review and editing (equal).

## Supporting information

Figure S1

Figure S2

Figure S3

Supplementary Material

## Data Availability

The data used for this study were deposited on the Dryad Digital Repositorey (https://doi.org/10.5061/dryad.2ngf1vhkd), following the data accessibility guidelines of Ecology and Evolution.
