# Supplementary material for: Tree litter functional diversity and nitrogen concentration enhance litter decomposition via changes in earthworm communities
Source: Ecol Evol. 2020 Jun 17;10(13):6752–68. doi: 10.1002/ece3.6474 (PMC7381558; doi:10.1002/ece3.6474)
Supplement: Supplementary file 4 — Supplementary Material [file ECE3-10-6752-s004.docx]

SUPPORTING INFORMATION FOR:

**Tree litter functional diversity and nitrogen concentration enhance litter decomposition via changes in earthworm communities**

Guillaume Patoine^1,2,^*, Helge Bruelheide^1,3^, Josephine Haase^4^, Charles Nock^4,5^, Niklas Ohlmann^4^, Benjamin Schwarz^6^, Michael Scherer-Lorenzen^4^, Nico Eisenhauer^1,2^

^1^ German Centre for Integrative Biodiversity Research (iDiv) Halle-Jena-Leipzig, Deutscher Platz 5e, 04103 Leipzig, Germany

^2^ Institute of Biology, Leipzig University, Deutscher Platz 5e, 04103 Leipzig, Germany

^3^ Institute of Biology/Geobotany and Botanical Garden, Martin Luther University Halle-Wittenberg, Am Kirchtor 1, 06108 Halle (Saale), Germany

^4^ Geobotany, Faculty of Biology, University of Freiburg, Schänzlestrasse 1, 79104 Freiburg, Germany

^5^ Department of Renewable Resources, Faculty of Agriculture, Life and Environmental Sciences, General Services Building, University of Alberta, Edmonton, Canada

^6^ Biometry and Environmental System Analysis, Faculty of Environment and Natural Resources, University of Freiburg, Tennenbacher Straße 4, 79106 Freiburg, Germany

* Corresponding author:

E-mail: guillaume.patoine@idiv.de

Address: German Centre for Integrative Biodiversity Research (iDiv) Halle-Jena-Leipzig, Deutscher Platz 5e, 04103 Leipzig, Germany

**Appendix 1: Supplementary Figures**


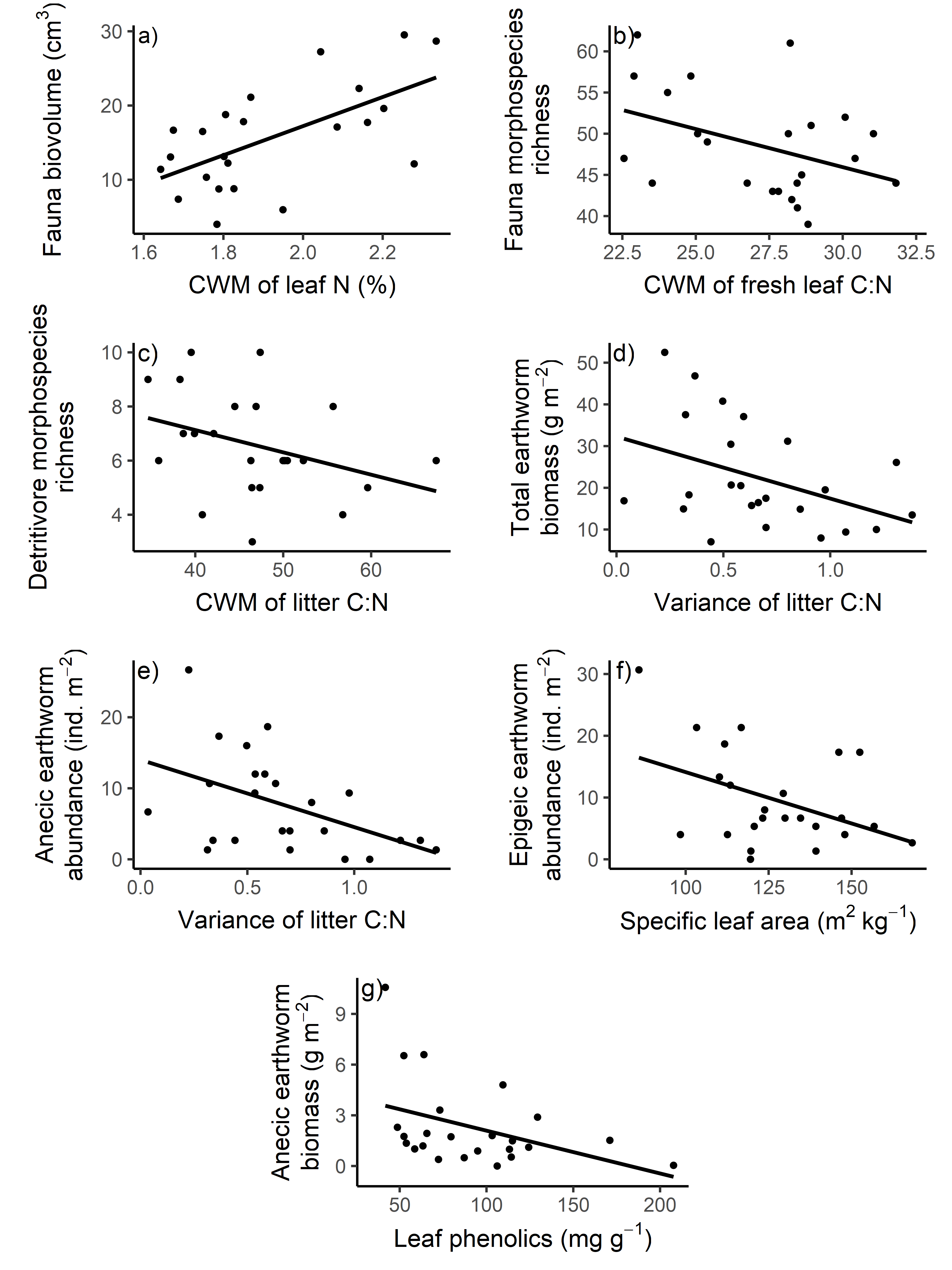


**Supplementary Figure 1.** a) Relationships between ground-dwelling fauna (from pitfall traps) biovolume and CWM of leaf N (Table 3). b) Influence of the CWM of leaf C:N on ground-dwelling fauna morphospecies richness, c) of CWM of litter C:N on detritivore morphospecies richness, d) of variance of litter C:N on total earthworm biomass and e) anecic earthworm abundance, f) of specific leaf area on epigeic earthworm abundance, and g) of leaf phenolic concentration on anecic earthworm biomass (Table 3). Lines are based on linear regressions.


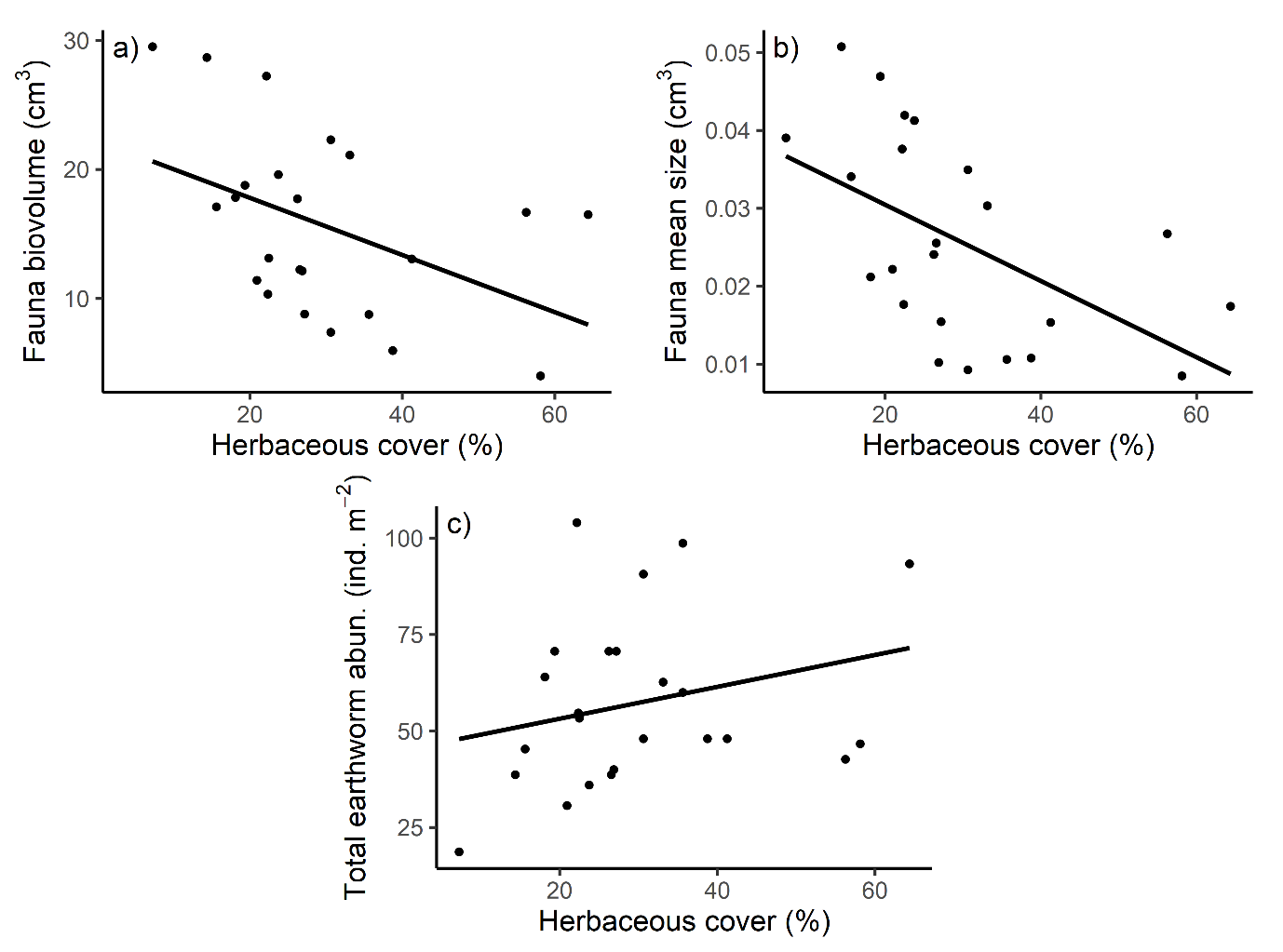


**Supplementary Figure 2.** Influence of herbaceous plant cover on a) ground-dwelling fauna biovolume and b) mean biovolume by specimen, and c) total earthworm abundance (Table 3). Lines are based on linear regressions.


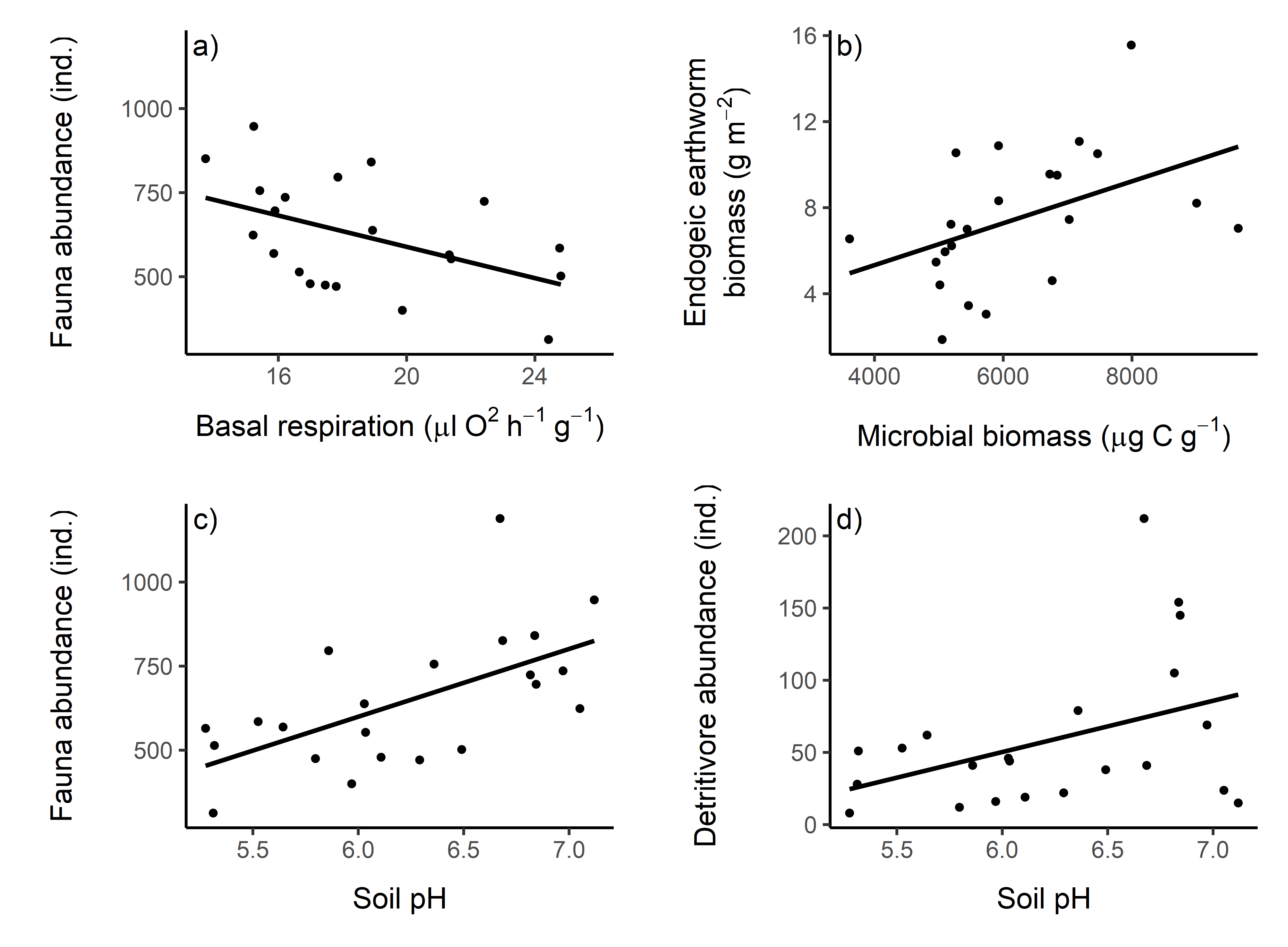


**Supplementary Figure 3.** Influence of a) soil basal respiration on ground-dwelling fauna abundance (individuals), b) of soil microbial biomass carbon on endogeic earthworm biomass, and of soil pH on c) ground-dwelling fauna abundance and d) detritivore abundance. Abundance are given as the mean number of individuals caught per pitfall trap over a period of 17 days. Five traps were used for each 43.5 m × 56.0 m plot. Lines are based on linear regressions.

**Appendix 2: Supplementary Tables**

**Supplementary Table 1.** Species pool at the BIOTREE-FD experiment.

| *Acer campestre* | *Pinus sylvestris* |
| --- | --- |
| *Acer platanoides* | *Populus tremula* |
| *Acer pseudoplatanus* | *Prunus avium* |
| *Betula pendula* | *Quercus petraea* |
| *Carpinus betulus* | *Sorbus aucuparia* |
| *Fagus sylvatica* | *Sorbus torminalis* |
| *Fraxinus excelsior* | *Tilia cordata* |
| *Larix decidua* | *Ulmus glabra* |

**Supplementary Table 2.** Number of retrieved litterbags for each BIOTREE-FD plot and litterbag mesh size. The three functional diversity (FD) indices used in this paper are provided for each plot. In total, it was possible to retrieve 167 from the initial 360 litterbags that had been installed

| **Plot** | **Litterbags retrieved** | | | **Tree FD** | **Litter FD, weighed** | **Litter FD, not weighed** |
| --- | --- | --- | --- | --- | --- | --- |
|  | **0.2 mm mesh** | **1 mm mesh** | **5 mm mesh** |  |  |  |
| 1 | 3 | 2 | 2 | 0.270 | 0.253 | 0.288 |
| 2 | 1 | 3 | 4 | 0.146 | 0.082 | 0.050 |
| 3 | 1 | 1 | 2 | 0.092 | 0.123 | 0.139 |
| 4 | 3 | 3 | 2 | 0.234 | 0.257 | 0.292 |
| 5 | 2 | 3 | 5 | 0.098 | 0.220 | 0.174 |
| 6 | 2 | 1 | 2 | 0.113 | 0.093 | 0.071 |
| 7 | 1 | 1 | 1 | 0.094 | 0.116 | 0.125 |
| 8 | 3 | 4 | 2 | 0.134 | 0.149 | 0.110 |
| 9 | 2 | 2 | 2 | 0.119 | 0.096 | 0.104 |
| 10 | 3 | 1 | 2 | 0.098 | 0.097 | 0.130 |
| 11 | 2 | 2 | 1 | 0.144 | 0.152 | 0.159 |
| 12 | 2 | 2 | 2 | 0.127 | 0.147 | 0.150 |
| 13 | 1 | 2 | 3 | 0.154 | 0.045 | 0.043 |
| 14 | 4 | 2 | 4 | 0.141 | 0.085 | 0.081 |
| 15 | 3 | 2 | 2 | 0.148 | 0.223 | 0.147 |
| 16 | 2 | 3 | 4 | 0.123 | 0.159 | 0.153 |
| 17 | 2 | 1 | 2 | 0.141 | 0.117 | 0.072 |
| 18 | 2 | 3 | 0 | 0.157 | 0.137 | 0.158 |
| 19 | 2 | 3 | 1 | 0.105 | 0.066 | 0.069 |
| 21 | 3 | 4 | 4 | 0.166 | 0.129 | 0.128 |
| 22 | 4 | 5 | 3 | 0.168 | 0.180 | 0.199 |
| 23 | 1 | 1 | 2 | 0.153 | 0.185 | 0.161 |
| 24 | 3 | 2 | 1 | 0.115 | 0.131 | 0.120 |
| 25 | 2 | 4 | 3 | 0.202 | 0.274 | 0.304 |

**Supplementary Table 3.** Number of morphospecies and specimens from pitfall traps samples and used for analysis.

| **Taxa** | **Level** | **Morphospecies** | **Specimens** |
| --- | --- | --- | --- |
| Isopoda | Order | 4 | 624 |
| Diplopoda | Class | 4 | 58 |
| Chilopoda | Class | 3 | 39 |
| Insecta | Class | 81 | 3,673 |
| Coleoptera | Order | 69 | 1,640 |
| Carabidae | Family | 32 | 992 |
| Staphylinidae | Family | 23 | 328 |
| Hymenoptera | Order | 5 | 1,938 |
| Formicidae | Family | 4 | 1,930 |
| Araneae | Order | 5 | 1,535 |
| Lycosidae | Family | 3 | 1,509 |
| Opiliones | Order | 4 | 92 |
| Acari | Subclass | 5 | 1,251 |
| Gastropoda | Class | 12 | 207 |
| Lumbricidae | Family | 3 | 10 |
| Collembola | Class | 4 | 7,204 |
| Larvae | Morph | 17 | 232 |
| **Total** |  | 142 | 14,925 |

**Supplementary Table 4.** Equations used for biovolume calculation and example taxa.

| **Geometric shape** | **Equation** | **Example taxa** |
| --- | --- | --- |
| Ellipsoid | $\frac{4}{3}*\pi*\frac{l}{2}*\frac{w}{2}*\frac{h}{2}$  $=\frac{\pi*lwh}{6}$ | Coleoptera, Araneae, Collembola |
| Semi-ellipsoid | $\frac{\frac{4}{3}*\pi*\frac{l}{2}*\frac{w}{2}*h}{2}$  $=\frac{\pi*lwh}{6}$ | Isopoda |
| Elliptic cylinder | $\pi*l*\frac{w}{2}*\frac{h}{2}$  $=\frac{\pi*lwh}{4}$ | Diplopoda, Lumbricidae, some larvae |

**Supplementary Table 5.** List of species traits used to calculate functional diversity (FD) indices and single trait metrics (community-weighted mean, CWM; trait variance, Var). Hypotheses are shown where trait CWM and Var are hypothesized to affect litter mass loss (LML) and soil fauna variables (abundance, richness, and biovolume) positively (+) or negatively (-).

| **Trait** | **Type** | **Scale/units** | **FD tree** | **FD litter** | **Hypotheses on LML** | | **Hypotheses on soil fauna** | |
| --- | --- | --- | --- | --- | --- | --- | --- | --- |
|  |  |  |  |  | **CWM** | **Var** | **CWM** | **Var** |
| Leaf phenology | Categorical | Evergreen (+) / Deciduous | x |  |  |  |  |  |
| Leaf type | Categorical | Coniferous (+) / Broadleaf |  | x |  |  |  |  |
| Light requirements as adults | Ordinal | Low, medium, high, very high | x |  |  |  |  |  |
| Mean annual stem growth | Ordinal | Low to high  (5 levels) | x |  |  |  |  |  |
| Crown architecture | Ordinal | Monopodial, sympodial-narrow, sympodial-broad | x |  |  |  |  |  |
| Root architecture | Ordinal | Shallow-rooted, heart-rooted, tap- rooted | x |  |  |  |  |  |
| Specific leaf area | Metric | m^2^ kg^-1^ | x |  | + |  | + |  |
| Leaf thickness | Metric | mm |  | x | - |  |  |  |
| Leaf toughness | Metric | N |  | x | - |  |  |  |
| Leaf N concentration | Metric | percent |  |  | + | + | + | + |
| Leaf C:N ratio | Metric | ratio | x |  | - | + | - | + |
| Litter C:N ratio | Metric | ratio |  | x | - | + | - | + |
| Leaf phenolic concentration | Metric | mg/g |  | x | - |  | - |  |
| Leaf tannin concentration | Metric | mg/g |  | x | - |  | - |  |

**Supplementary Table 6.** Significance of results of linear mixed effects models testing the effects of litter and tree functional dispersion, the CWM of six traits, the variance (Var) of three traits, soil basal respiration (resp.) and microbial biomass, herbaceous cover, and soil pH on the abundance (Abun.), morphospecies richness (Rich.), and biovolume (Biovol.) of total fauna, detritivores and isopods from pitfall traps, and the abundance and biomass of all earthworms (total), and epigeic, anecic, and endogeic earthworms separately. We used experimental block as random factor. Only significant and marginally significant effects (p < 0.05 and p < 0.1 respectively) are reported in the table. Cells with significant values are filled. Cell fill and text color represents a positive effect for blue and a negative effect for red.

|  |  | **Total fauna** | | | **Detritivores** | | | **Isopods** | | | **Total earthworms** | | **Anecic earthworms** | | **Epigeic earthworms** | | **Endogeic earthworms** | |
| --- | --- | --- | --- | --- | --- | --- | --- | --- | --- | --- | --- | --- | --- | --- | --- | --- | --- | --- |
|  |  | **Abun.** | **Rich.** | **Biovol.** | **Abun.** | **Rich.** | **Biovol.** | **Abun.** | **Rich.** | **Biovol.** | **Abun.** | **Biom.** | **Abun.** | **Biom.** | **Abun.** | **Biom.** | **Abun.** | **Biom.** |
| **Functional dispersion** | Tree community |  |  |  |  |  |  |  |  |  |  |  |  |  |  |  |  |  |
|  | Litter |  |  |  |  |  |  |  |  |  |  |  |  |  | 0.045 | 0.020 |  |  |
| **Trait CWM** | Leaf N |  | 0.065 | 0.007 |  |  |  |  |  |  |  |  |  |  |  |  |  |  |
|  | Leaf C:N |  | 0.049 | 0.014 |  |  |  |  |  |  |  |  |  |  |  |  |  |  |
|  | Litter C:N | 0.071 |  |  | 0.071 | 0.001 |  | 0.099 |  |  |  |  |  |  |  |  |  |  |
|  | SLA |  |  |  |  |  |  |  |  |  | 0.060 |  |  |  | 0.029 | 0.005 |  |  |
|  | Leaf phenolics |  |  |  |  |  |  |  |  |  |  |  |  |  |  | 0.036 |  |  |
|  | Leaf tannins |  |  |  |  |  |  |  |  |  |  |  |  |  |  | 0.070 |  |  |
| **Trait variance** | Leaf N |  |  |  |  |  |  |  |  | 0.099 |  |  |  |  |  |  |  |  |
|  | Leaf C:N |  |  |  |  |  |  |  |  |  |  |  |  |  |  |  |  |  |
|  | Litter C:N |  |  |  |  |  |  |  |  |  |  | 0.036 | 0.010 | 0.012 |  |  |  |  |
| **Soil Microbes** | Basal resp. | 0.016 |  |  |  |  |  |  |  |  |  |  |  |  |  |  |  |  |
|  | Biomass C |  |  |  |  |  |  |  |  |  |  |  |  |  |  |  | 0.088 | 0.027 |
| **Herb layer** | Herb cover (%) |  |  | 0.023 |  |  |  |  |  |  | 0.048 | 0.071 |  |  |  |  |  |  |
| **Soil** | pH | 0.001 |  |  | 0.009 |  | 0.043 |  |  | 0.056 | 0.052 |  |  |  |  |  | 0.075 |  |

**Supplementary Table 7.** Results of initial structural equation model on the effects of litter functional diversity (FD) and litter C:N on epigeic and anecic earthworm abundance (abun.) and litter mass loss (LML) in litterbags of three mesh sizes (as illustrated in Supplementary figure 5). Given are unstandardized path coefficients (estimate), standard error of regression weights (S.E.), standardized path coefficients (Std. Estim.), degrees of freedom (df), and level of significance (p). Significant relationships (p < 0.05) are bold and marginally significant relationships (p < 0.1) are in italic.

| **Response** | **Predictor** | **Estimate** | **S.E.** | **Std. Estimate** | **p** |
| --- | --- | --- | --- | --- | --- |
| **Epigeic earthwom abun.** | **Litter FD** | **53.704** | **23.313** | **0.429** | **0.021** |
| Epigeic earthwom abun. | Litter C:N | -0.268 | 0.270 | -0.185 | 0.321 |
| Anecic earthworm abun. | Litter FD | -3.715 | 21.934 | -0.035 | 0.866 |
| Anecic earthworm abun. | Litter C:N | 0.255 | 0.254 | 0.206 | 0.314 |
| *LML, 5 mm* | *Litter FD* | *65.529* | *37.254* | *0.371* | *0.079* |
| LML, 5 mm | Litter C:N | -0.505 | 0.405 | -0.248 | 0.211 |
| LML, 5 mm | Epigeic earthworm abun. | -0.141 | 0.300 | -0.100 | 0.639 |
| LML, 5 mm | Anecic earthworm abun. | 0.314 | 0.319 | 0.191 | 0.325 |
| LML, 1 mm | Litter FD | 21.644 | 30.704 | 0.145 | 0.481 |
| *LML, 1 mm* | *Litter C:N* | *-0.591* | *0.333* | *-0.343* | *0.076* |
| LML, 1 mm | Epigeic earthworm abun. | 0.162 | 0.247 | 0.136 | 0.514 |
| LML, 1 mm | Anecic earthworm abun. | -0.169 | 0.263 | -0.121 | 0.521 |
| LML, 0.2 mm | Litter FD | 2.984 | 31.416 | 0.015 | 0.924 |
| **LML, 0.2 mm** | **Litter C:N** | **-0.757** | **0.341** | **-0.334** | **0.027** |
| **LML, 0.2 mm** | **Epigeic earthworm abun.** | **0.758** | **0.253** | **0.484** | **0.003** |
| *LML, 0.2 mm* | *Anecic earthworm abun.* | *-0.493* | *0.269* | *-0.270* | *0.067* |
| Litter FD | Litter C:N | 0.026 | 0.070 | 0.078 | 0.708 |
|  |  |  |  |  |  |
| Correlations |  |  |  |  |  |
| **LML, 5 mm** | **LML, 1 mm** | **40.834** | **19.153** | **0.496** | **0.033** |
| LML, 5 mm | LML, 0.2 mm | 5.922 | 17.598 | 0.070 | 0.736 |
| LML, 1 mm | LML, 0.2 mm | 2.788 | 14.479 | 0.040 | 0.847 |
